# Use of PROMIS® to screen for depression in children with arthritis

**DOI:** 10.1186/s12969-020-00482-1

**Published:** 2020-11-23

**Authors:** Yufan Yan, Karen L. Rychlik, Marc B. Rosenman, Michael L. Miller

**Affiliations:** 1 Feinberg School of Medicine, Northwestern University, Chicago, IL USA; 2 Stanley Manne Children’s Research Institute, Lurie Children’s Hospital, Chicago, IL USA; 3 Department of Pediatrics, Feinberg School of Medicine, Northwestern University, Chicago, IL USA; 4 Mary Ann & J. Milburn Smith Child Health Research, Outreach and Advocacy Center, Ann & Robert H. Lurie Children’s Hospital, Chicago, IL USA; 5 Division of Rheumatology, Department of Pediatrics, Feinberg School of Medicine, Northwestern University, Chicago, IL USA

**Keywords:** Juvenile idiopathic arthritis, Health related quality of life, Depression, Anxiety

## Abstract

**Background:**

Children with JIA may experience difficulty with health related quality of life (HRQOL). The Patient Reported Outcomes Measurement Information System (PROMIS) a patient related outcome (PRO) measure, covers HRQOL domains that include physical function, mental health, and social interactions. During initial use, we found PROMIS identified children with symptoms of depression, sometimes before they shared those feelings with parents or members of the clinic team. We studied the use of PROMIS for this purpose, and to determine what demographic, clinical, and other characteristics might be related to higher depressive symptom scores.

**Methods:**

From March 2014 – February 2017, at each visit, all JIA patients having met ILAR classification criteria seen by M.L.M. received the PROMIS Short Form 35 v.1.0, as part of routine care. T scores were calculated from raw scores for mobility, anxiety, depressive symptoms, fatigue, peer relationships, and pain interference domains. Data extracted by optical mark recognition software were merged with electronic medical record (EMR data), extracted by Extract/Transform/Load software, including joint counts, visit age, ANA, RF, and HLA-B27 status. Mixed effects models were used to identify significant associations of independent variables with depression T scores.

**Results:**

Data from 148 patients were analyzed (114 females for 435 visits, 34 males for 118 visits; 13.8 ± 2.8 years): 70 persistent oligoarthritis, 9 extended oligoarthritis, 19 ERA, 21 polyarthritis (RF-), 5 polyarthritis (RF+), 11 undifferentiated arthritis, 3 psoriatic arthritis, 10 systemic arthritis). T scores showed wide ranges within individual JIA categories, with similar mean scores for all groups. Univariate linear mixed effects models showed significant relationships to depression T scores of gender and race (males and Asian patients with lower T scores, *p* < .0001, *p* = 0.091, respectively), joint count (*p* = 0.002), pain interference score (*p* = 0.0004), and Patient and Physician Global Assessment (*p* = 0.004, *p* < .0001, respectively). No particular JIA category was associated with Depression T scores. HRQOL domains were interrelated (p < .0001), including patients reporting symptoms of depression tending also to report symptoms of anxiety. PROMIS identified 15 patients who did not otherwise report depressive symptoms, but needed referral for counseling; eight did not endorse depressive symptoms until the 2nd or 3rd visit. Only 3 patients had disease flare. Concerns besides arthritis such as parental conflict or school bullying were elicited in 7 patients during interviews with the social worker. All patients expressed being worried about their arthritis.

**Conclusion:**

PROMIS is useful in screening JIA patients for symptoms of depression, particularly to identify patients who might not otherwise report these symptoms. The other PROMIS domain scores are related to reporting of symptoms of depression, as is Patient and Physician Global Assessment. Future studies will use PROMIS questionnaires incorporated into the EMR, permitting data entry by tablets and an online patient portal. This will make possible comparisons of HRQOL in children with JIA to those with other chronic rheumatic and non-rheumatic diseases.

**Supplementary Information:**

The online version contains supplementary material available at 10.1186/s12969-020-00482-1.

## Background

Juvenile Idiopathic Arthritis (JIA) is characterized by chronic arthritis; manifestations such as number of joints, presence of fever, and types of complications vary according to JIA category [1]. Children with JIA are at risk of developing long-term disability from continued disease activity and may experience difficulty with health related quality of life (HRQOL) and [2]. JIA has been associated with such HRQOL related problems as emotional difficulties (social isolation, depression, and anxiety), pain, and decreased physical function [3, 4]. Problems with HRQOL have been associated with more severe pain and more limited mobility [4]. In one study, psychiatric disorders were diagnosed almost three times more frequently in children with JIA group compared to control patients, often with depression [5]. In another study, more than one fifth of JIA patients reported “feeling depressed”, which correlated with higher levels of pain, disease activity, and reduced mobility [6]. Depression, but not anxiety, was among the best predictors for HRQOL in JIA patients [7] and strongly correlated with pain intensity [8].

HRQOL in children with arthritis has been assessed by patient reported outcome (PRO) measures, including questionnaires covering physical function, Childhood Health Assessment Questionnaire (CHAQ) for [9] and Juvenile Arthritis Functional Assessment Scale (JAFAS) [10], and measures such as the Patient Reported Outcomes Measurement Information System (PROMIS) [11]. PROMIS, a Patient Reported Outcome (PRO) measure developed by the National Institutes of Health (www.healthmeasures.net) as a suite of PROs for adults and children, covers HRQOL domains that include physical function, mental health, and social interactions. While PROMIS measures implemented in EMRs permit additional questions to be asked, conditioned upon responses to initial banks of questions (using item response theory), paper based versions offer potential for rapid screening, particularly when EMR implementation is still being scoped and planned. During a pilot phase, we found that the paper based initial pediatric version of PROMIS identified children with symptoms of depression, sometimes before they shared those feelings with parents or members of the clinic team. We were interested in finding out whether HRQOL assessment given during an extended period to all JIA patients at every visit might assist in early recognition of patients with symptoms of depression. In this hypothesis generating study, we studied the use of PROMIS for this purpose, and to determine what demographic, clinical, and other characteristics might be related to higher depressive symptom scores.

## Methods

### Patients

All patients, ages 8–17, having previously met International League of Associations for Rheumatology (ILAR) classification criteria for JIA [9] who were seen in rheumatology clinic by the senior author MLM between March 2014 – February 2017 received the HRQOL questionnaire PROMIS (validated for ages 8–17; see below) at each visit, unless they had severe cognitive impairment (4 patients). All diagnoses were reviewed and verified by other members of the Division of Rheumatology. Demographic, disease characteristics, and HRQOL data were extracted for all visits of these patients during this time period. Data were extracted and analyzed after obtaining Lurie Children’s IRB approval of extracting data without need for consent. Only the senior author who provided care for patients had access to identified data. Data were de-identified prior to analysis.

### Demographic and disease characteristics

Age data (birth date, date of each visit) were extracted. Additional extractable disease characteristics consisted of diagnostic category, joint count (active joints, as defined by Petty et al. [12], pain score, medications, Physician and Patient Global Assessment. Data for anti-nuclear antibody (ANA), rheumatoid factor (RF), and HLA-B27 status obtained within the first 4 months of diagnosis were extracted.

### Physician and patient assessment

Physician Global Assessment of overall disease activity used a scale from 0 to 10. Patient pain was rated on a Likert scale in response to the question “By giving a number between 0 and 10, with 0 being no pain and 10 being the worst possible pain, how much pain on average have you experienced from your arthritis over the past week?” Patient Global Assessment was rated on a Likert scale in response to the question “By giving a number between 0 and 10, with 0 being doing very poorly and 10 being doing very well, how have you experienced your arthritis in general over the past week? Include not only pain, but also how you feel about your arthritis, how having arthritis affects your getting along with family and friends, and how well you can move around.”

### Outcome measures

At every visit, all patients answered Patient Reported Outcomes Medical Information System questionnaire (PROMIS v1.0 Pediatric Profile 25, healthmeasures.net). PROMIS is a National Institutes of Health project to create item banks across a variety of adult and pediatric diseases to assess patient reported outcomes. PROMIS was selected for its coverage of important domains of pediatric patient-reported outcomes, efficiency of administration, and availability for clinical care without requiring licensing fees. The version used was paper based; it does not use sets of banks of questions made available by computer, depending on specific prior responses (item response theory) but has been validated for children ages 8–17 years [11]. This version was used throughout the study period. The questionnaire, given as soon as each patient had vital signs taken, entered the exam room, but before the nurse or pediatric rheumatologist (MLM) entered, took no more than 15 min to complete. Prior to subsequent scoring, results were examined by one of the authors (M.L.M.) during each patient’s visit, to identify children (based on answering at least one depressive symptom with “often” or “almost always”) for immediate interview by the team’s social worker, to assess need for referral for mental health services. On the PROMIS v1.0 Pediatric Profile 25, children completed four questions in each of six domains (labeled physical function – mobility, anxiety, depressive symptoms, fatigue, peer relationships, and pain interference) and asked to rank pain intensity on a Likert Scale with values of 0 through 10 (the Asthma impact scale included in the original validation study was not included in the version used in this study). Each item asks the children themselves about symptoms in the past 7 days before filling out the questionnaire and has five response categories ranging from either “never” to “almost always” or, “from with no trouble” to “not able to do.” Responses are scored ranging from 1 to 5 where a higher score represents more frequent or severe symptom. Raw scores for each domain and the entire questionnaire are the sum of the values of the response to each question within each domain. Lower scores indicate fewer problems for five domains (physical function – mobility, anxiety, depression, fatigue, and pain interference). Lower scores indicated more problems for the domain peer relationships. .

For this study, the PROMIS Pediatric Profile Scoring Manual (see Appendix B) provided tables for calculating scores as T scores, rescaled into standardized scores with mean of 50 and standard deviation of 10. T scores are referenced in the manual to a healthy cohort. PROMIS currently provides the same scoring steps, but only as automated scoring through a data collection tool (https://www.assessmentcenter.net/ac_scoringservice).

### Data entry into the electronic medical record

Since July 2008, all patient visits have been documented in an EMR (EpicCare™, Epic Systems, Verona, WI). Starting in 2010, a discrete data structure (called flow sheet rows in the Epic EMR) for JIA patients has been incorporated into the EMR. All patients with JIA have joint examination and related clinical data entered as discrete data into these flow sheet rows, as part of routine care for each outpatient encounter.

### Data extraction from the PROMIS questionnaire and from the electronic medical record

Using forms formatted for optical mark recognition (Appendix A), paper questionnaires were scanned into pdf files; data from the pdf files were extracted by optical mark recognition software (Remark Office OMR®, Gravic, Inc.). The questionnaire forms had space for patient labels containing bar codes that identified the specific date of the visit, allowing questionnaires to be scanned into each patient’s EMR and for questionnaire data to be merged with EMR data, using Extract/Transform/Load software, for all visits March 2014 – February 2017, including active joint counts, visit age, ANA, RF, and HLA-B27 status, as described [13].

### Statistical methods

Mixed effects models were used to identify significant associations of independent variables with depression T scores, the primary outcome variable. Descriptive statistics are reported with frequencies and percentages for categorical variables and means and standard deviations for continuous data, including all PROMIS scores. A series of linear mixed effects models were run with each independent variable to determine the relationship with the primary outcome; random subject effect was included to account for correlations of measurements within patients who had multiple observations. All covariate categories were compared to a reference group, consistently chosen as the largest category within the variable. Diagnoses of Polyarthritis (Rheumatoid Factor Negative), Polyarthritis (Rheumatoid Factor Positive), and Extended Oligoarthritis were combined into a “Polyarthritis Group” for analyses. Significant independent variables were chosen for a final multivariable model using associations with a *p* < 0.15. To eliminate collinearity from highly correlated independent variables, only variables with the strongest statistically significant relationship with the primary outcome were used. In the instance of the two highly correlated Global Assessments by both patient and physician, the patient Global Assessment was used in the final model. Since PROMIS domain scores were highly correlated with each other, the Anxiety score was used for the final model. The final linear mixed effects model includes all parameters with associations of *p* < 0.05. Missing data for two Global Assessment measures were < 8% of total visits; for all other variables, < 1% of data were missing. Analyses were for complete cases only. All *p*-values are unadjusted, all test are two-sided and analyses were performed using SAS version 9.4 (SAS Institute Inc., Cary, NC).

## Results

### Patients

Data from 148 patients (114 females for 435 visits; 34 males for 118 visits; 13.8 ± 2.8 years) were analyzed: 70 persistent oligoarthritis, 9 extended oligoarthritis, 19 enthesitis related arthritis (ERA), 21 polyarthritis (RF), 5 RF), 11 undifferentiated arthritis, 3 psoriatic arthritis, 10 systemic arthritis (Table 1). Data for anti-nuclear antibody (ANA), rheumatoid factor (RF), and HLA-B27 status obtained within the first 4 months of diagnosis were and available (as noted in parentheses) for the JIA categories as follows: 10 systemic arthritis patients (5 ANA, 0 RF, 0 HLA-B27); 70 persistent oligoarthritis patients (22 ANA, 1 RF [negative on repeat test more than 3 months later], 1 HLA-B27); 9 extended oligoarthritis patients (6 ANA, 1 RF+ [negative on repeat more than 3 months later], 1 HLA-B27); 21 polyarthritis (RF negative) patients (11 ANA+, 0 RF+, 0 HLA-B27+); 5 polyarthritis (RF+) patients (4 ANA+, 5 RF+, 0 HLA-B27+); 3 psoriatic arthritis patients (1 ANA+, 0 RF+, 1 HLA-B27+), 19 enthesitis related arthritis patients (7 ANA+, 0 RF+, 8 HLA-B27)+. Except for the patient with persistent oligoarthritis, all RF positive patients had confirmation on repeat testing at least 3 months afterwards. Seven patients received glucocorticosteroids.

**Table 1:** Demographic and disease characteristics

	All	Systemic	Psoriatic	Poly RF+	Poly RF-	Oligo	Extended Oligo	ERA	Other
N patients (visits)	148 (553)	10 (48)	3 (21)	5 (20)	21 (108)	70 (228)	9 (65)	19 (63)	11 (28)
Age in years (mean ± SD)	13.8 ± 2.8	12.3 ± 2.0	15.1 ± 1.5	15.9 ± 1.3	12.7 ± 2.9	13.4 ± 2.6	13.7 ± 2.6	16.2 ± 1.8	15.6 ± 2.6
Gender: F/M	114/34	7/3	2/1	4/1	17/4	56/14	9/0	13/6	6/5
Years followed (mean ± SD)	4.5 ± 3.3	5.5 ± 3.1	5.0 ± 4.4	4.4 ± 3.2	5.0 ± 2.9	4.0 ± 3.3	5.4 ± 2.8	2.9 ± 1.6	6.1 ± 5.3
% ANA+	41%	50%	33%	80%	52%	31%	67%	37%	36%
% RF+	5%	0%	0%	100%	0%	1%^a^	11%	0%	0%
% B27+	11%	0%	33%	0%	0%	9%	11%	42%	0%
Race/Ethnicity (%)									
White/non-Hispanic	45.3%	70.0%	0%	20.0%	38.1%	45.7%	55.6%	47.4%	45.5%
Hispanic	34.5%	30.0%	66.7%	60.0%	47.6%	34.3%	33.3%	26.3%	9.1%
African-American	13.5%	0%	0%	20.0%	4.8%	12.9%	11.1%	21.0%	27.2%
Asian	5.4%	0%	33.3%	0%	9.5%	4.3%	0%	5.3%	18.2%
Other	1.4%	0%	0%	0%	0%	2.9%	0%	0%	0%

Legend: ^a^found on a single visit only; not present on repeat > 3 months later

### HRQOL domain scores

T scores derived from raw scores showed wide ranges within individual JIA categories, with similar mean scores for all groups (Table 2). Analysis for all visits for the entire group of patients of domain T scores for association with the primary outcome, depression T scores was then performed.

**Table 2:** Raw and T-scores for PROMIS domains

	All	Systemic	Psoriatic	Poly RF+	Poly RF-	Oligo	Extended Oligo	ERA	Other
Mobility									
raw	3.5 ± 3.7	2.9 ± 3.8	5.2 ± 3.5	3.1 ± 4.2	3.1 ± 2.9	3.4 ± 3.7	3.0 ± 4.0	4.0 ± 3.6	4.5 ± 4.4
T	26.9 ± 6.6	25.7 ± 6.8	30.0 ± 5.8	26.1 ± 7.9	26.3 ± 5.2	26.8 ± 6.6	26.2 ± 7.4	27.8 ± 6.2	29.1 ± 8.6
Anxiety									
raw	3.3 ± 3.9	3.1 ± 3.4	4.9 ± 3.9	4.2 ± 3.6	3.7 ± 4.3	2.8 ± 3.8	3.6 ± 3.9	3.6 ± 4.3	4.1 ± 3.1
T	44.9 ± 10.9	44.3 ± 9.7	49.4 ± 10.4	47.3 ± 10.1	45.6 ± 11.9	43.3 ± 10.6	46.0 ± 10.9	45.2 ± 11.9	47.5 ± 8.5
Depression									
raw	2.2 ± 3.2	2.8 ± 3.0	3.4 ± 3.0	2.9 ± 3.7	2.7 ± 3.9	1.6 ± 2.8	2.2 ± 3.1	1.9 ± 3.4	2.3 ± 2.8
T	44.7 ± 9.0	47.0 ± 8.6	48.9 ± 8.5	46.8 ± 9.9	46.1 ± 10.2	43.2 ± 8.1	45.2 ± 8.5	43.6 ± 9.4	45.3 ± 8.4
Fatigue									
raw	4.2 ± 4.1	2.9 ± 3.2	6.0 ± 4.0	3.9 ± 4.2	4.5 ± 4.3	4.0 ± 4.1	4.1 ± 4.2	4.7 ± 4.3	5.4 ± 2.9
T	48.5 ± 10.8	45.2 ± 8.8	53.1 ± 10.2	47.1 ± 11.6	49.0 ± 11.5	47.9 ± 10.8	48.3 ± 10.8	49.7 ± 11.1	52.2 ± 7.5
Relationship									
raw	13.3 ± 3.3	12.8 ± 3.5	15.2 ± 1.2	13.1 ± 3.0	12.3 ± 4.1	13.5 ± 3.1	13.0 ± 2.9	14.0 ± 3.2	14.2 ± 2.0
T	52.4 ± 8.8	51.1 ± 9.4	57.7 ± 4.8	50.1 ± 7.6	50.0 ± 10.3	52.8 ± 8.4	50.1 ± 7.9	54.7 ± 8.7	54.2 ± 6.6
Pain Interference									
raw	5.2 ± 4.7	3.1 ± 3.6	7.5 ± 5.1	4.7 ± 4.4	4.9 ± 4.8	5.3 ± 4.9	5.3 ± 4.6	6.0 ± 4.6	5.5 ± 4.1
T	50.0 ± 10.5	45.6 ± 8.6	54.4 ± 11.3	48.7 ± 10.0	49.3 ± 10.8	50.2 ± 10.8	50.5 ± 10.3	51.9 ± 10.2	51.1 ± 8.8

### Univariate linear mixed effects models of HRQOL domain scores

Table 3 summarizes the series of univariate linear mixed effects models for each independent variable. Male visits are more likely to have a lower PROMIS depression T score of more than 4 points than female visits (coefficient estimate = − 4.08 (95% confidence intervals: − 7.18, − 0.97), *p* < .0001). As the number of joint count increases by one, the depression score increases by an estimate of 0.36 points ((95% CI: 0.14, 0.60), *p* = 0.002). When the patient pain score increases by one point the depression score also increases by an estimate of 0.35 ((95% CI: 0.12, 0.58) *p* = 0.004). While the Physician Global Assessment score increases one point the depression score will increase by an estimate of 0.49 (95% CI: 0.22, 0.76), *p* = 0.0004), however, an increase of one point for the Patient Global Assessment score decreases depression by an estimate of − 1.16((95% CI: − 1.48, − 0.84), *p* < .0001). All PROMIS scores significantly predict the depression score (p < .0001), and as they increase by one point, depression increases by the Anxiety estimates of 0.59 (95% CI: 0.54, 0.64), Mobility estimate of 0.36 (95% CI: 0.25, 0.46), Pain Interference estimate of 0.30 (95% CI: 0.23, 0.37), Physical Function estimate = 0.34 (95% CI: 0.28, 0.41), and as Peer Relationships increases (signifying better relationships) depression score decreases by an estimate of − 0.26 (95% CI: − 0.34, − 0.19). Race, Hispanic ethnicity and diagnoses, along with ANA, RF, and HLA-B27 status, medications taken, and age of visit were not significantly predictive of the PROMIS depression T score.

**Table 3:** Patient Characteristics in Linear Mixed Effects Models

Independent Variables	Coefficient Estimate (Standard Error)	*p*-value	95% Confidence Intervals
Gender			
Male	−4.08 (1.58)	<.0001	(−7.18, −0.97)
Female	reference		
Race			
Asian	−4.96 (2.93)	0.091	(−10.73, 0.81)
Black	−0.99 (2.08)	0.635	(−5.08, 3.11)
Other	1.1 (1.34)	0.409	(−1.52, 3.73)
White	reference		
Hispanic Ethnicity			
Hispanic	1.31 (1.43)	0.359	(−1.50, 4.12)
Not Hispanic	reference		
Diagnosis			
Enthesitis	−0.46 (1.73)	0.793	(−3.86, 2.95)
Other	0.66 (2.29)	0.772	(−3.83, 5.16)
Psoriatic	2.55 (2.97)	0.391	(−3.28, 8.38)
Polyarthritis Group	1.9 (1.37)	0.166	(−0.79, 4.59)
Systemic	−1.11 (2.77)	0.688	(−6.55, 4.33)
Oligo	reference		
ANA Present	0.01 (1.12)	0.99	(−2.19, 2.22)
ANA Absent	reference		
RF Present	−0.37 (2.32)	0.874	(4.93, 4.20)
RF Absent	reference		
HLA-B27 Present	− 1.15 (1.74)	0.508	(−4.58, 2.27)
HLA-B27 Absent	reference		
Age at Visit	−0.02 (0.19)	0.926	(−0.39, 0.36)
Joint Count	0.36 (0.12)	0.002	(0.14, 0.60)
Patient Pain Score	0.35 (0.12)	0.004	(0.12, 0.58)
Patient Global Assessment	−1.16 (0.16)	<.0001	(−1.48, −0.84)
Physician Global Assessment	0.49 (0.14)	0.0004	(0.22, 0.76)
Anxiety PROMIS	0.59 (0.03)	<.0001	(0.54, 0.64)
Mobility PROMIS	0.36 (0.05)	<.0001	(0.25, 0.46)
Pain Interference PROMIS	0.3 (0.04)	<.0001	(0.23, 0.37)
Peer Relationships PROMIS	−0.26 (0.04)	<.0001	(− 0.34, − 0.19)
Physical Function PROMIS	0.34 (0.03)	<.0001	(0.28, 0.41)

### Multivariable linear mixed effects model of HRQOL domain scores

A final multivariable linear mixed effects model was constructed and is shown in Table 4. Asian patient visits were significant (estimate = − 3.71 (95% CI: − 6.88, − 0.54), *p* = 0.022) and can be interpreted as a decrease in the depression score of close to 4 points for Asian patient visits. As the Patient Global Assessment increases by one point, depression score decreases by half a point (estimate = − 0.5 (95% CI: − 0.75, − 0.25), *p* = .0001) and as the PROMIS Anxiety score increases by one point, depression increases by an estimate of 0.57 ((95% CI: 0.52, 0.63), *p* < .0001).

**Table 4:** Multivariable Linear Mixed Effects Model

Independent Variables	Coefficient Estimate (Standard Error)	*p*-value	95% Confidence Intervals
Intercept	22.96 (1.87)	<.0001	(19.26, 26.66)
Race			
Asian	−3.71 (1.61)	0.022	(−6.88, −0.54)
Black	−1.44 (1.14)	0.209	(−3.68, 0.81)
Other	0.12 (0.78)	0.877	(−1.41, 1.65)
White	reference		
Patient Global Assessment	−0.5 (0.13)	0.0001	(−0.75, − 0.25)
Anxiety PROMIS	0.57 (0.03)	<.0001	(0.52, 0.63)

### Screening by PROMIS to recognize children needing referral for mental health services

The rheumatology team’s social worker was asked to interview 31 patients, based solely on answers to symptoms of depression, as described in Methods. Of these, 15 were assessed as needing referral for mental health services (Table 5). Seven of these patients answered affirmatively to depressive symptoms at the first visit during the study period; however, 8 patients did not endorse depressive symptoms until the 2nd or 3rd visit. Although most patients had polyarticular disease, this was not statistically significant. Most patients complained of arthralgia; only 3 patients had disease flare at the time they reported depressive symptoms. Concerns besides arthritis such as parental conflict or school bullying were elicited in 7 patients during interviews with the social worker. All patients expressed being worried about their arthritis.

**Table 5:** JIA patients referred for counseling based on answers to PROMIS questions about symptoms of depression

Visit during study period	Months since 1st study period visit	Age at visit	Gender	JIA Category	Arthralgia present	Arthritis flare	Joint count	Patient Global Assessment	Physician Global Assessment	Subsequent concerns expressed
1st	0	8.6	F	Oligo-p	yes	no	0	10	1	
1st	0	8.8	F	Poly RF-	yes	yes (severe)	10	3	7	sibling conflict
1st	0	9.4	F	Oligo-p	yes	yes (mild)	2	N/A	3	
1st	0	13.1	F	Oligo-p	yes	no	0	5	1	parents’ divorce
1st	0	14.6	F	Poly RF-	yes	no	0	4	1	
1st	0	15	F	Oligo-p	no	no	0	N/A	0	
1st	0	16.1	M	Poly RF-	no	no	0	10	1	
2nd	8.7	8.7	F	Poly RF-	yes	no	0	2	1	father’s recent CVA
3rd	4.1	9.3	F	Poly RF-	yes	yes (severe)	17	5	9	school bullying
2nd	9.0	14	F	Poly RF-	no	no	0	3	2	conflict with parents and siblings
2nd	2.8	15.6	M	Poly RF-	no	no	0	5	1	
2nd	9.9	16.2	F	Poly RF+	no	no	0	10	2	parents’ marital conflict
3rd	27.8	16.3	F	ERA	yes	yes (mild)	0	7	4	
3rd	6.1	16.5	F	ERA	yes	yes	4	4.5	7	
3rd	16.5	16.9	F	Poly RF-	yes	no	0	8	4	school bullying

Legend: *Oligo-p* persistent oligoarthritis; *Poly RF-* Polyarthritis (Rheumatoid Factor Negative); *Poly RF+* Polyarthritis (Rheumatoid Factor Positive), *ERA* Enthesitis Related Arthritis

## Discussion

Children with arthritis may have problems in several HRQOL domains, including symptoms of depression and anxiety [5], decreased mobility, and pain [4]; clinic visits are important for detecting these problems. We conducted this study to examine the extent to which PROMIS, a PRO measure of HRQOL, could assist in identifying children in need of counseling for mood related symptoms, as well as to determine the relationship of demographic and clinical data and of other HRQOL domains to symptoms of depression.

Mixed effects models confirmed our clinical expectations. We found that patient assessment of overall well being as affected by arthritis and Physician Global Assessment of disease activity were associated with reporting symptoms of depression. We also found that HRQOL domains are themselves interrelated, characterized by the finding that patients reporting symptoms of depression tended also to report symptoms of anxiety. Gender differences are similar to those previously reported [reviewed in reference [3]. We found that reporting of pain interfering with daily activities was associated with reporting symptoms of depression, similar to previous studies showing pain itself to be associated with depressive symptoms [3, 4, 6]. Another domain associated with increased depressive symptoms was difficulty with peer relationships (the wording of questions in PROMIS results in inverse scoring). Our finding that no particular JIA category was associated with depression T scores also matches our clinical experience. While we thought it might play a role in worsening symptoms of depression, there were too few patients receiving glucocorticosteroids to be able to determine this. There was no association of any other medications with depression T scores.

Our anticipation that PROMIS would help screen patients for clinically significant symptoms of depression was confirmed by our finding that we were able to use PROMIS as an additional screening tool. During the study period, 15 patients were referred for counseling, based solely on responses to PROMIS. Most did not indicate severe symptoms until after the first visit during the study period. This may be a result either of PROMIS itself, which is not a formal instrument for diagnosis of depression, but screens for symptoms. However, later detection may also reflect the need for clinicians to assess at each visit whether JIA patients are depressed, because of the cumulative stressors they experience. It is possible that some children feel more comfortable with initially indicating symptoms of depression in writing, since PROMIS led to discovery during social worker evaluation of previously unrecognized stressors such as parental conflict or school bullying.

Peer relationships are an important aspect of development, and found to be related to both pain interference and depressive symptom HRQOL domains in JIA. Studies have shown that children who suffer from chronic pain may also have poorer peer relationships [5]. In a qualitative study of adolescents with JIA, patients reported that their peers had negative attitudes towards their pain, which made them feel sad, angry, and left out [11]. Three studies examining quality of life in children and adolescents with chronic arthritis found that social activities were negatively affected by arthritic pain; the extent of impact depended on disease severity [14–16].

This hypothesis generating study had several limitations. We included data for each patient visit over the course of 3 years, but patients did not have an equal number of visits. To address the possibility that interpretation of the data could skew towards patients who had more visits, we used mixed effects models to control for the dependence within the data of varying numbers of visits for individual patients. Another limitation was that there were fewer male than female patients in all categories; age, gender, and JIA category distribution in our population is comparable to previously published descriptions. Our studying every patient seen over a 3 year period prevented selection bias. In our study patients had T scores similar to those of the reference population used for validation of PROMIS scoring [12]. This may be a result of decreased sensitivity of the PROMIS instrument used in detecting problems with HRQOL domains in JIA patients. Another possibility is that JIA patients with more severe problems with HRQOL are a minority. However, identifying these patients remains important for optimizing care of their chronic conditions, and we found PROMIS plays a useful role in this assessment.

We used the first pediatric short form version of PROMIS available, throughout the study period. While this permitted every patient seen to complete a questionnaire, use of subsequent versions with greater numbers of questions would have permitted use of cut-points for mobility, fatigue, pain interference, and upper extremity function domains, established by Morgan et al. in their validation study of PROMIS in JIA [17]. However, the version we used fit well with clinic work flows, resulting in being able to have every patient at every visit fill out the questionnaire. Studies using later versions of PROMIS, in which data is collected at set intervals, perhaps using mobile applications, when patients are not necessarily in clinic, would help determine whether the findings in this study are reproduced. This approach to HRQOL evaluation may also become increasingly important in an era in which telemedicine visits will be increasingly used.

## Conclusion

This study found PROMIS useful in screening JIA patients for symptoms of depression, including those who had not otherwise reported these symptoms. The other PROMIS domain scores are related to reporting of symptoms of depression, as is Patient and Physician Global Assessment. Future studies will use PROMIS questionnaires incorporated into the EMR, permitting data entry by tablets and an online patient portal. This will make possible comparisons of HRQOL in children with JIA to those with other chronic rheumatic and non-rheumatic diseases.

## Supplementary Information

**Additional file 1 Appendix A**. PROMIS Pediatric Profile 25 Survey OMR version.

**Additional file 2 Appendix B**. PROMIS Pediatric Profile Scoring Manual.

## Data Availability

The datasets are not publicly available as they contain identifiable information.

## Abbreviations

ANA: Anti-nuclear antibody
CHAQ: Child Health Assessment Questionnaire
EMR: Electronic medical record
ERA: Enthesitis related arthritis
HRQOL: Health related quality of life
ILAR: International League of Associations for Rheumatology
JAFAS: Juvenile Arthritis Functional Assessment Scale
JIA: Juvenile idiopathic arthritis
PROMIS: Patient Reported Outcomes Medical Information System
RF: Rheumatoid factor
