# Supplementary material for: Use of PROMIS® to screen for depression in children with arthritis
Source: Pediatr Rheumatol Online J. 2020 Nov 23;18:92. doi: 10.1186/s12969-020-00482-1 (PMC7686667; doi:10.1186/s12969-020-00482-1)
Supplement: Supplementary file 1 — Additional file 1 Appendix A. PROMIS Pediatric Profile 25 Survey OMR version. [file 12969_2020_482_MOESM1_ESM.pdf]

Ann & Robert H. Lurie Children's Hospital of Chicago
Chicago, IL 60611
PROMIS Pediatric Profile 25 Survey

P T R H

Medical Record No.
Patient Name
Birthdate
Physician
Please align patient label to the right

To be completed by/for children aged 8-17 yrs

Directions: Please fill in marks like this: ● **Not like this:** ✓ ⊗

Physical Function - Mobility

**In the past 7 days…**	With No Trouble	With a little trouble	With some trouble	With a lot of trouble	Not able to do so
1. I could do sports and exercise that other kids my age could do	O	O	O	O	O
2. I could get up from the floor	O	O	O	O	O
3. I could walk upstairs without holding on to anything	O	O	O	O	O
4. I have been physically able to do the activities I enjoy most	O	O	O	O	O

Anxiety

**In the past 7 days…**	Never	Almost Never	Some times	Often	Almost Always
1. I felt like something awful might happen	O	O	O	O	O
2. I felt nervous	O	O	O	O	O
3. I felt worried	O	O	O	O	O
4. I was afraid that I would make mistakes	O	O	O	O	O

Depressive Symptoms

**In the past 7 days…**	Never	Almost Never	Some times	Often	Almost Always
1. I felt everything in my life went wrong	O	O	O	O	O
2. I felt lonely	O	O	O	O	O
3. I felt sad	O	O	O	O	O
4. I thought that my life was bad	O	O	O	O	O

Investigator Format@2008-2012 PROMIS Health Organization and PROMIS Cooperative Group

Fatigue

**In the past 7 days…**	Never	Almost Never	Some times	Often	Almost Always
1. Being tired made it hard to keep up with my schoolwork	O	O	O	O	O
2. I got tired easily	O	O	O	O	O
3. I was too tired to do sports or exercise	O	O	O	O	O
4. I was too tired to enjoy the things I like to do	O	O	O	O	O

Peer Relationships

**In the past 7 days…**	Never	Almost Never	Some times	Often	Almost Always
1. I felt accepted by other kids my age	O	O	O	O	O
2. I was able to count on my friends	O	O	O	O	O
3. My friends and I helped each other out	O	O	O	O	O
4. Other kids wanted to be my friend	O	O	O	O	O

Pain Interference

**In the past 7 days…**	Never	Almost Never	Some times	Often	Almost Always
1. I had trouble sleeping when I had pain	O	O	O	O	O
2. It was hard for me to pay attention when I had pain	O	O	O	O	O
3. It was hard for me to run when I had pain	O	O	O	O	O
4. It was hard for me to walk one block when I had pain	O	O	O	O	O

Pain Intensity

**In the past 7 days…**	No Pain										Worst pain you can imagine
How bad was your pain on average?	0	1	2	3	4	5	6	7	8	9	10

Signature: ____

Relationship to patient: ______________________________________

Date/Time: __________________ Interpreter (as applicable): ____________________

Investigator Format@2008-2012 PROMIS Health Organization and PROMIS Cooperative Group
