# Supplementary material for: Use of PROMIS® to screen for depression in children with arthritis
Source: Pediatr Rheumatol Online J. 2020 Nov 23;18:92. doi: 10.1186/s12969-020-00482-1 (PMC7686667; doi:10.1186/s12969-020-00482-1)
Supplement: Supplementary file 2 — Additional file 2 Appendix B. PROMIS Pediatric Profile Scoring Manual. [file 12969_2020_482_MOESM2_ESM.pdf]

Patient-Reported Outcomes Measurement Information System
Dynamic Tools to Measure Health Outcomes From the Patient Perspective

PROMIS PEDIATRIC PROFILE INSTRUMENTS

A brief guide to the PROMIS Profile instruments for pediatric respondents:

PROMIS-25 Profile v1.0
PROMIS-37 Profile v1.0
PROMIS-49 Profile v1.0

ABOUT PROMIS PROFILES

PROMIS Pediatric Profile instruments are a collection of short forms containing a fixed number of items from six PROMIS domains (depressive symptoms, anxiety, mobility, pain interference, fatigue, and peer relationships) along with a single item on pain intensity. There are three profile lengths - the PROMIS-25 includes 4 items per domain; the PROMIS-37 includes 6 items per domain; the PROMIS-49 includes 8 items per domain. As with other PROMIS instruments, the Profiles are generic rather than disease-specific. They assess all domains over the past seven days except for physical function which has no timeframe specified.

These PROMIS Pediatric Profile instruments are intended for pediatric self-report (ages 8-17).

In selecting between PROMIS Pediatric Profile Instruments, the difference is instrument length. The reliability and precision of the short forms within a domain is highly similar. The PROMIS-25 Profile short forms are labeled “4a”. The PROMIS-37 Profile short forms are labeled “6a” and the PROMIS-49 Profile short forms are labeled “8a”.

PROFILE ITEM SELECTION

The short forms that make up the PROMIS Profile Instruments include “high information” items. The selection of items was in part based on item rankings within each domain using two psychometric criteria: (1) maximum interval information, and 2) CAT simulations. Item rankings were similar for both criteria. For the maximum interval criterion, each item information function was integrated (without weighting) for the interval from the mean to 2 SDs worse than the mean. For the CAT simulations, responses to all items in each bank were generated using a random sample of 1,000 simulees drawn separately for each bank (centered on 0.5 SD worse than the general population mean). Items were rank ordered based on their average administration rank over the simulees. Content experts reviewed the items and rankings and made cuts of 4, 6, and 8 items. The items within each instrument are nested/overlap (e.g., the 8-item form is the 6-item form plus two additional items).

ADMINISTRATION INSTRUCTIONS

The PROMIS Profile Instruments are administered as short forms (not adaptive administration). Short forms are ideal when clinical researchers prefer to ask the same question of all respondents or of the same respondent over time to enable a more direct comparability across people or time. Instruct participants to answer all of the items (i.e., questions or statements) presented.

SCORING THE INSTRUMENT

Scoring for PROMIS instruments uses Item Response Theory (IRT), a family of statistical models that link individual items to a presumed underlying trait or concept represented by all items in the item bank. In the case of the PROMIS Profiles, the instrument is made up of seven individual short forms that are scored individually. The single pain intensity item is not scored but reported as its raw score (e.g., 0 to 10). Scoring uses item-level calibrations. This means that the most accurate way to score a PROMIS Profile is to utilize scoring tools within Assessment Center that look at responses to each item for each participant. We refer to this as "response pattern scoring." Data collected in Assessment Center automatically generates response pattern scoring. The response pattern scoring tools within Assessment Center can be used even if data was collected on paper or in another software package if data is entered into a launched study. Because response pattern scoring is more accurate than the use of raw score/scale score look up tables, it is preferred. However, if you aren't able to use response pattern scoring, you can use the instructions below which rely on raw score/scale score look-up tables.

Each item has five response options ranging in value from 0 to 4, except for the pain intensity item which has eleven response options ranging in value from 0 to 10. A raw score is created from each short form that makes up the Profile. To find the total raw score for a short form with all questions answered, sum the values of the response to each question within each domain. For example, for the 25-item Profile, the lowest possible raw score within anxiety is 0 (a score of 0 on all four items); the highest possible raw score is 16 (see all short form scoring tables in Appendix).

For the PROMIS-37 and PROMIS-49, scores can be approximated if a participant skips a question. If items are missing, first check how many items were answered. For both short forms with 6 and 8 items, confirm that 4 items were answered per short form. After confirming that enough responses were provided, sum the response scores from the items that were answered for that given short form (depressive symptoms, anxiety, mobility, pain interference, fatigue, and peer relationships). Multiply this sum by the total number of items in the short form (6 or 8). Finally, divide by the number of items that were answered. For example, if a respondent answered 5 of 8 questions and answered all items with the second lowest response option (2), you would sum all responses (10), multiply by the number of items in the short form (8) and divide by the number of items that were answered (5). Here (10x8)/5=16. If the result is a fraction, round up to the nearest whole number. This is a pro-rated raw score.

Again, the formula is:

$$\frac{\text{(Raw sum x number of items on the short form)}}{\text{Number of items that were actually answered}}$$

Locate the applicable score conversion table in the Appendix and use this table to translate the total raw score or pro-rated score into a T-score for each short form for each participant. The T-score rescales the raw score into a standardized score with a mean of 50 and a standard deviation (SD) of 10. Therefore a person with a T-score of 40 is one SD below the mean. The standardized T-score is reported as the final score for each participant.

Here is an example. For the PROMIS-25 Profile instrument, an anxiety raw score of 10 converts to a T-score of 62.42 with a standard error (SE) of 5.14 (see scoring table for the Anxiety 4a short form in appendix). Thus, the 95% confidence interval around the observed score ranges from 52.4 to 72.5 (T-score ± (1.96*SE) or 62.42 ± (1.96*5.14).

For pro-rated scores, this calculation assumes that responses are missing at random. This isn't always true. Therefore, use caution when interpreting the final pro-rated T-score.

The PROMIS Pediatric Profile domains (depressive symptoms, anxiety, mobility, pain interference, fatigue, peer relationships), a score of 50 is the average for the calibration sample with a standard deviation of 10. The calibration sample which was enriched for chronic illness compared to a general population sample. A score of 50 likely represents somewhat sicker people than the general population. The T-score is provided with an error term (Standard Error or SE). The Standard Error is a statistical measure of variance and represents the "margin of error" for the T-score.

All of the Profile instruments include a single pain intensity item. This item is not scored using Item Response Theory. Instead, raw responses (0-10) can be used in analyses.

Important: *A higher PROMIS T-score represents more of the concept being measured.* For negatively-worded concepts like anxiety, a T-score of 60 is one SD worse than average. By comparison, an anxiety T-score of 40 is one SD better than average. However, for positively-worded concepts like physical function, a T-score of 60 is better than average while a T-score of 40 is better.

STATISTICAL CHARACTERISTICS

There are four key features of the score for PROMIS Profile instruments:

- **Reliability**: The degree to which a measure is free of error. It can be estimated by the internal consistency of the responses to the measure, or by correlating total scores on the measure from two time points when there has been no true change in what is being measured (for z-scores, reliability = 1 – SE^2).
- **Precision**: The consistency of the estimated score (reciprocal of error variance).
- **Information:** The precision of an item or multiple items at different levels of the underlying continuum (for z-scores, information = 1/SE^2).
- **Standard Error (SE):** The possible range of the actual final score based upon the scaled T-score. For example, with a T-score of 52 and a SE of 2, the 95% confidence interval around the actual final score ranges from 48.1 to 55.9 (T-score ± (1.96*SE) = 52 ± 3.9 = 48.1 to 55.9).

The final score is represented by the T-score, a standardized score with a mean of 50 and a standard deviation (SD) of 10.

PREVIEW OF SAMPLE ITEM

Figure 1 shows a PROMIS Profile Pediatric Anxiety item as it would appear to a study participant during data collection in Assessment Center. Several formats for presenting the items are available for computer-based administration through Assessment Center (see FAQ section).

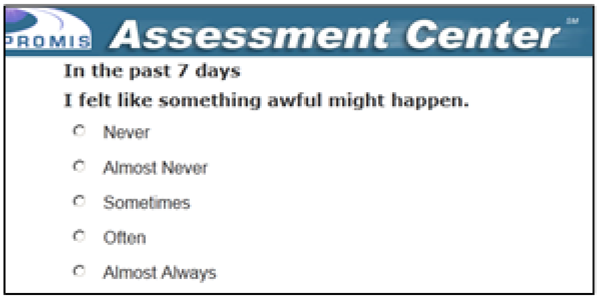

Figure 1

Figure 2 is an excerpt from the paper version of the PROMIS-25 Pediatric Profile instrument. This is the paper version format used for all Profile instruments. Note that there are participant and investigator versions of the Profile PDFs. The Investigator version is shown in Figure 2. The Participant version does not have the item IDs and response scores. This minimizes the visual clutter a participant sees when completing a

PROMIS Profile on paper. The investigator version helps the clinical researcher identify how to identify and score items.

	Anxiety<br>In the past 7 days…	Never	Almost Never	Sometimes	Often	Almost Always
2220R2<br>5	I felt like something awful might happen..	☐ 0	☐ 1	☐ 2	☐ 3	☐ 4
713R1<br>6	I felt nervous..	☐ 0	☐ 1	☐ 2	☐ 3	☐ 4

Figure 2

FREQUENTLY ASKED QUESTIONS (FAQ)

Q: *I am interested in learning more. Where can I do that?*

All instruments are available through Assessment Center, which houses all PROMIS instruments for each domain. Assessment Center is a free online research management tool. It enables researchers to create study-specific websites for capturing participant data securely. Studies can include measures within the Assessment Center library, as well as custom instruments created or entered by the researcher. PROMIS instruments (short forms, CATs, profiles) are a central feature of the instrument library within Assessment Center. Any PROMIS measure can be included in an online study or downloaded for administration on paper.

Detailed statistical information and development history about PROMIS items and instruments are available for review at nihpromis.org or assessmentcenter.net. To learn more, contact help@assessmentcenter.net.

Q: *Do I need to register with PROMIS to use these instruments?*

Yes, to get a copy of these instruments, we ask that you register with Assessment Center and endorse the PROMIS Terms and Conditions of Use, so that we are better able to track who has accessed instruments for research. Assessment Center is available at assessmentcenter.net.

Q: *Are these instruments available in other languages?*

These instruments will be available in Spanish in Assessment Center. The PROMIS group is working to translate Profiles into other languages. Information on available translations is updated periodically at http://nihpromis.org/measures/translations.

Q: How do I handle multiple responses when administering a short form on paper?

Guidelines on how to deal with multiple responses have been established. Resolution depends on the responses noted by the research participant.

- If two or more responses are marked by the respondent, and they are next to one another, then a data entry specialist will be responsible for randomly selecting one of them to be entered and will write down on the form which answer was selected. *Note: To randomly select one of two responses, the data entry specialist will flip a coin (heads - higher number will be entered; tails – lower number will be entered).To randomly select one of three (or more) responses, a table of random numbers should be used with a statistician's assistance.*

- If two or more responses are marked, and they are NOT all next to one another, the response will be considered missing.

Q: *What is the minimum change on a PROMIS instrument that represents a clinically meaningful difference?*

This question is related to an area of active research in the PROMIS network, namely the determination of the "minimally important difference" or "MID" for a PROMIS instrument. A manuscript in the *Journal of Clinical Epidemiology* outlines the process for MIDs for adult PROMIS measures and estimates the MIDs for six PROMIS-Cancer scales: Yost, K. J., Eton, D. T., Garcia, S. F., & Cella, D. (2011). Minimally important differences were estimated for six PROMIS-Cancer scales in advanced-stage cancer patients. *Journal of Clinical Epidemiology, 64(5)*, 507-16.

As described in that manuscript, the MID is a tool to enhance the interpretability of patient-reported outcomes and is often defined as the "the smallest difference in score in the domain of interest which patients perceive as beneficial and which would mandate, in the absence of troublesome side effects and excessive cost, a change in the patient's management" (Jaeschke R, Singer J, Guyatt GH. Measurement of health status. Ascertaining the minimal clinically important difference. *Controlled Clinical Trials* 1989; 10(4):407-415).

APPENDIX-SCORING TABLES

PROMIS – 25

Anxiety 4a
Short Form Conversion Table

Raw Score	T-score	SE*
0	34.5	6.3
1	39.5	5.5
2	42.6	5.4
3	45.6	5.2
4	48.2	5.1
5	50.6	5.1
6	53.1	5.1
7	55.4	5.1
8	57.8	5.1
9	60.1	5.1
10	62.4	5.1
11	64.8	5.1
12	67.2	5.2
13	69.8	5.1
14	72.4	5.2
15	75.2	5.2
16	78.7	5.2

* SE = Standard error

Depressive Symptoms 4a
Short Form Conversion Table

Raw Score	T-score	SE*
0	37.7	6.4
1	43.5	5.1
2	46.7	4.9
3	49.7	4.6
4	52.1	4.4
5	54.3	4.3
6	56.3	4.2
7	58.3	4.2
8	60.2	4.2
9	62.0	4.2
10	63.9	4.2
11	65.8	4.2
12	67.8	4.2
13	69.9	4.2
14	72.1	4.3
15	74.6	4.4
16	78.0	4.7

* SE = Standard error

Fatigue 4a
Short Form Conversion Table

Raw Score	T-score	SE*
0	35.4	6.5
1	40.7	5.6
2	44.2	5.4
3	47.2	5.2
4	49.8	5.1
5	52.2	5.0
6	54.4	5.0
7	56.5	4.9
8	58.6	4.9
9	60.6	4.9
10	62.7	4.9
11	64.7	4.9
12	66.9	4.9
13	69.1	4.9
14	71.5	5.0
15	74.1	5.0
16	77.7	5.2

* SE = Standard error

Mobility 4a
Short Form Conversion Table

Raw Score	T-score	SE*
0	20.0	4.5
1	23.1	4.1
2	25.1	3.9
3	26.9	3.9
4	28.4	3.8
5	30.0	3.8
6	31.5	3.8
7	32.9	3.8
8	34.4	3.8
9	36.0	3.8
10	37.6	3.9
11	39.3	4.1
12	41.2	4.4
13	42.9	4.2
14	45.5	4.4
15	48.9	4.7
16	57.1	7.0

* SE = Standard error

Pain Interference 4a
Short Form Conversion Table

Raw Score	T-score	SE*
0	36.7	6.1
1	42.0	4.9
2	44.4	4.8
3	47.2	4.4
4	49.3	4.3
5	51.3	4.1
6	53.2	4.1
7	55.0	4.0
8	56.7	4.0
9	58.4	4.0
10	60.1	4.0
11	61.8	4.0
12	63.6	4.1
13	65.5	4.1
14	67.7	4.2
15	70.0	4.3
16	74.0	5.0

* SE = Standard error

Peer Relationships 4a
Short Form Conversion Table

Raw Score	T-score	SE*
0	22.9	5.1
1	25.7	4.8
2	27.7	4.7
3	29.8	4.5
4	31.7	4.5
5	33.6	4.4
6	35.4	4.4
7	37.2	4.4
8	38.9	4.4
9	40.7	4.4
10	42.6	4.5
11	44.5	4.6
12	46.7	4.8
13	48.9	4.7
14	51.9	5.1
15	55.3	5.4
16	61.1	6.6

* SE = Standard error

Pediatric version

PROMIS – 37

Anxiety 6a<br>*Short Form Conversion Table*		
Raw Score	T-score	SE*
0	33.5	6.0
1	38.2	5.1
2	40.9	4.9
3	43.5	4.6
4	45.6	4.5
5	47.6	4.3
6	49.5	4.3
7	51.3	4.2
8	53.0	4.2
9	54.6	4.2
10	56.3	4.2
11	57.8	4.2
12	59.4	4.2
13	61.0	4.2
14	62.6	4.2
15	64.2	4.2
16	65.8	4.1
17	67.4	4.1
18	69.1	4.1
19	70.8	4.2
20	72.6	4.2
21	74.4	4.2
22	76.5	4.3
23	78.7	4.3
24	81.4	4.3
* SE = Standard error		

Depressive Symptoms 6a<br>*Short Form Conversion Table*		
Raw Score	T-score	SE*
0	36.9	6.1
1	42.4	4.8
2	45.3	4.5
3	47.8	4.1
4	49.9	3.9
5	51.7	3.7
6	53.3	3.5
7	54.8	3.4
8	56.2	3.4
9	57.6	3.4
10	58.9	3.3
11	60.2	3.3
12	61.5	3.3
13	62.8	3.3
14	64.0	3.3
15	65.3	3.3
16	66.6	3.3
17	67.9	3.3
18	69.2	3.3
19	70.6	3.3
20	72.1	3.4
21	73.6	3.4
22	75.4	3.6
23	77.4	3.8
24	80.4	4.1
* SE = Standard error		

Fatigue 6a<br>*Short Form Conversion Table*		
Raw Score	T-score	SE*
0	32.8	5.9
1	37.5	5.1
2	40.4	4.9
3	43.0	4.6
4	45.2	4.5
5	47.2	4.4
6	49.1	4.3
7	50.8	4.3
8	52.5	4.2
9	54.1	4.2
10	55.7	4.2
11	57.3	4.2
12	58.8	4.2
13	60.4	4.2
14	61.9	4.2
15	63.4	4.2
16	65.0	4.2
17	66.6	4.2
18	68.2	4.2
19	69.9	4.2
20	71.6	4.2
21	73.5	4.3
22	75.6	4.4
23	77.9	4.4
24	80.8	4.5
* SE = Standard error		

Pediatric version

PROMIS – 37

Mobility 6a		
Short Form Conversion Table		
Raw Score	T-score	SE*
0	17.1	3.8
1	19.5	3.8
2	21.3	3.7
3	22.8	3.6
4	24.2	3.5
5	25.4	3.5
6	26.6	3.4
7	27.8	3.4
8	28.9	3.3
9	30.0	3.3
10	31.1	3.3
11	32.2	3.3
12	33.3	3.3
13	34.4	3.3
14	35.5	3.4
15	36.7	3.4
16	37.9	3.5
17	39.2	3.5
18	40.6	3.6
19	42.1	3.8
20	43.9	4.1
21	45.7	4.1
22	48.1	4.3
23	51.4	4.8
24	58.4	6.7
* SE = Standard error		

Pain Interference 6a		
Short Form Conversion Table		
Raw Score	T-score	SE*
0	35.0	5.7
1	39.9	4.5
2	42.0	4.3
3	44.3	3.9
4	46.0	3.7
5	47.7	3.5
6	49.2	3.5
7	50.6	3.4
8	51.9	3.3
9	53.2	3.3
10	54.4	3.3
11	55.7	3.3
12	56.9	3.3
13	58.1	3.3
14	59.3	3.3
15	60.5	3.3
16	61.8	3.3
17	63.1	3.3
18	64.4	3.3
19	65.7	3.3
20	67.2	3.4
21	68.7	3.5
22	70.6	3.7
23	72.5	3.8
24	76.1	4.5
* SE = Standard error		

Peer Relationships 6a		
Short Form Conversion Table		
Raw Score	T-score	SE*
0	19.7	4.5
1	22.2	4.3
2	23.8	4.3
3	25.6	4.1
4	27.1	4.0
5	28.6	3.9
6	30.0	3.9
7	31.4	3.8
8	32.8	3.8
9	34.1	3.8
10	35.4	3.8
11	36.7	3.8
12	38.1	3.8
13	39.4	3.8
14	40.7	3.8
15	42.1	3.8
16	43.6	3.9
17	45.1	3.9
18	46.7	4.0
19	48.4	4.1
20	50.3	4.3
21	52.4	4.4
22	55.0	4.7
23	58.0	5.1
24	63.2	6.2
* SE = Standard error		

Pediatric version

PROMIS – 49

Anxiety 8a
Short Form Conversion Table

Raw Score	T-score	SE*
0	32.3	5.7
1	36.7	4.9
2	39.2	4.7
3	41.4	4.3
4	43.3	4.2
5	45.1	4.0
6	46.7	3.9
7	48.2	3.8
8	49.6	3.8
9	50.9	3.7
10	52.3	3.7
11	53.5	3.7
12	54.8	3.7
13	56.0	3.7
14	57.3	3.7
15	58.5	3.7
16	59.7	3.7
17	60.9	3.7
18	62.1	3.7
19	63.3	3.7
20	64.5	3.7
21	65.8	3.7
22	67.0	3.7
23	68.3	3.7
24	69.6	3.7
25	70.9	3.7
26	72.3	3.7
27	73.7	3.7
28	75.2	3.8
29	76.8	3.9
30	78.6	4.0
31	80.5	4.0
32	82.8	3.9

* SE = Standard error

Depressive Symptoms 8a
Short Form Conversion Table

Raw Score	T-score	SE*
0	35.2	5.8
1	40.4	4.6
2	43.2	4.2
3	45.5	3.8
4	47.4	3.6
5	49.1	3.4
6	50.5	3.3
7	51.9	3.2
8	53.2	3.1
9	54.3	3.1
10	55.5	3.0
11	56.6	3.0
12	57.7	3.0
13	58.7	3.0
14	59.7	2.9
15	60.7	2.9
16	61.8	2.9
17	62.8	2.9
18	63.7	2.9
19	64.7	2.9
20	65.7	2.9
21	66.8	2.9
22	67.8	2.9
23	68.8	2.9
24	69.9	2.9
25	70.9	2.9
26	72.1	3.0
27	73.2	3.0
28	74.5	3.1
29	75.9	3.2
30	77.5	3.4
31	79.3	3.5
32	81.9	3.7

* SE = Standard error

Fatigue 8a
Short Form Conversion Table

Raw Score	T-score	SE*
0	31.1	5.6
1	35.3	4.8
2	38.0	4.5
3	40.3	4.3
4	42.3	4.1
5	44.1	4.0
6	45.7	3.9
7	47.2	3.8
8	48.7	3.8
9	50.1	3.8
10	51.4	3.7
11	52.7	3.7
12	54.0	3.7
13	55.3	3.7
14	56.5	3.7
15	57.8	3.7
16	59.0	3.7
17	60.2	3.7
18	61.5	3.7
19	62.7	3.7
20	63.9	3.7
21	65.2	3.7
22	66.4	3.7
23	67.7	3.7
24	69.0	3.7
25	70.4	3.7
26	71.8	3.7
27	73.2	3.8
28	74.8	3.8
29	76.4	3.9
30	78.3	4.0
31	80.3	4.0
32	82.8	3.9

* SE = Standard error

Pediatric version

PROMIS – 49

Mobility 8a<br>*Short Form Conversion Table*		
Raw Score	T-score	SE*
0	15.2	3.0
1	17.1	3.1
2	18.6	3.1
3	19.9	3.1
4	21.1	3.0
5	22.2	2.9
6	23.2	2.9
7	24.2	2.8
8	25.1	2.8
9	25.9	2.7
10	26.8	2.7
11	27.6	2.7
12	28.4	2.7
13	29.2	2.7
14	30.0	2.7
15	30.9	2.7
16	31.7	2.7
17	32.5	2.7
18	33.3	2.7
19	34.2	2.7
20	35.0	2.8
21	36.0	2.8
22	36.9	2.9
23	37.9	3.0
24	39.0	3.1
25	40.1	3.2
26	41.4	3.3
27	42.8	3.5
28	44.4	3.9
29	46.1	3.9
30	48.4	4.2
31	51.6	4.8
32	58.5	6.7
* SE = Standard error		

Pain Interference 8p<br>*Short Form Conversion Table*		
Raw Score	T-score	SE*
0	34.0	5.6
1	38.7	4.4
2	40.6	4.2
3	42.7	3.8
4	44.3	3.7
5	45.8	3.4
6	47.1	3.3
7	48.4	3.2
8	49.5	3.2
9	50.6	3.1
10	51.7	3.1
11	52.7	3.1
12	53.7	3.0
13	54.7	3.0
14	55.7	3.0
15	56.6	3.0
16	57.6	3.0
17	58.5	3.0
18	59.5	3.0
19	60.4	3.0
20	61.4	3.0
21	62.4	3.0
22	63.4	3.0
23	64.4	3.0
24	65.4	3.1
25	66.5	3.1
26	67.6	3.2
27	68.8	3.2
28	70.1	3.3
29	71.5	3.4
30	73.2	3.7
31	75.0	3.8
32	78.0	4.3
* SE = Standard error		

Peer Relationships 8a<br>*Short Form Conversion Table*		
Raw Score	T-score	SE*
0	17.7	4.0
1	20.0	3.9
2	21.4	3.9
3	23.0	3.7
4	24.4	3.7
5	25.7	3.6
6	26.9	3.5
7	28.1	3.4
8	29.2	3.4
9	30.4	3.4
10	31.4	3.3
11	32.5	3.3
12	33.6	3.3
13	34.6	3.3
14	35.6	3.3
15	36.7	3.3
16	37.7	3.3
17	38.8	3.3
18	39.8	3.3
19	40.9	3.3
20	42.0	3.3
21	43.1	3.4
22	44.3	3.4
23	45.5	3.4
24	46.7	3.5
25	48.0	3.5
26	49.4	3.6
27	50.9	3.7
28	52.6	3.9
29	54.5	4.1
30	56.8	4.5
31	59.5	4.8
32	64.4	6.0
* SE = Standard error		

Pediatric version
